# Polyphenol oxidase as a biochemical seed defense mechanism

**DOI:** 10.3389/fpls.2014.00689

**Published:** 2014-12-10

**Authors:** E. Patrick Fuerst, Patricia A. Okubara, James V. Anderson, Craig F. Morris

**Affiliations:** ^1^Department of Crop and Soil Sciences, Washington State UniversityPullman, WA, USA; ^2^Root Disease and Biological Control Research Unit, United States Department of Agriculture – Agricultural Research Service, Washington State UniversityPullman, WA, USA; ^3^Biosciences Research Laboratory, United States Department of Agriculture – Agricultural Research ServiceFargo, ND, USA; ^4^Western Wheat Quality Laboratory, United States Department of Agriculture – Agricultural Research Service, Washington State UniversityPullman, WA, USA

**Keywords:** seed defense, seed decay, seed longevity, weed seed bank

## Abstract

Seed dormancy and resistance to decay are fundamental survival strategies, which allow a population of seeds to germinate over long periods of time. Seeds have physical, chemical, and biological defense mechanisms that protect their food reserves from decay-inducing organisms and herbivores. Here, we hypothesize that seeds also possess enzyme-based biochemical defenses, based on induction of the plant defense enzyme, polyphenol oxidase (PPO), when wild oat (*Avena fatua* L.) caryopses and seeds were challenged with seed-decaying *Fusarium* fungi. These studies suggest that dormant seeds are capable of mounting a defense response to pathogens. The pathogen-induced PPO activity from wild oat was attributed to a soluble isoform of the enzyme that appeared to result, at least in part, from proteolytic activation of a latent PPO isoform. PPO activity was also induced in wild oat hulls (lemma and palea), non-living tissues that cover and protect the caryopsis. These results are consistent with the hypothesis that seeds possess inducible enzyme-based biochemical defenses arrayed on the exterior of seeds and these defenses represent a fundamental mechanism of seed survival and longevity in the soil. Enzyme-based biochemical defenses may have broader implications since they may apply to other defense enzymes as well as to a diversity of plant species and ecosystems.

## SEED SURVIVAL AND LONGEVITY

Seed decay has been defined as “A process in which the physical integrity of a seed is degraded, ultimately leading to death” (Long et al., 2014). Seed dormancy and resistance to decay are fundamental survival strategies which allow a population of seeds to germinate over time both within and across years (Baskin and Baskin, 2006; Dalling et al., 2011; Long et al., 2014). Thus, seed dormancy and resistance to decay are core components of plant population ecology across a diversity of natural- and agro-ecosystems. The nutritional reserves of plant seeds are obviously in demand by a great diversity of herbivores and microorganisms. Therefore defenses against such organisms are required for seed survival in the soil.

**FIGURE 1:**
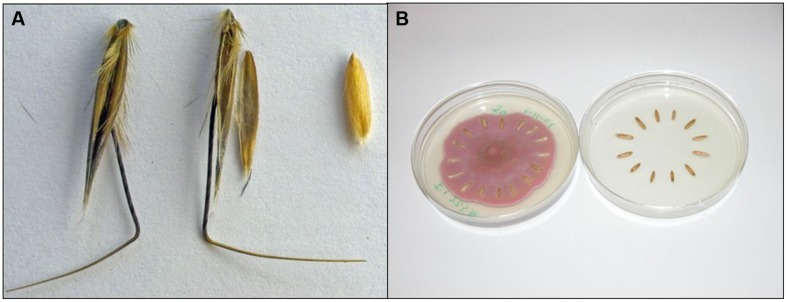
**Seeds and pathogen: experimental materials. (A)** Wild oat isoline ‘M73’: seed (left), dissected (middle) into lemma with awn, and palea, and caryopsis (right). We refer to lemma and palea as “hulls.” When we refer to wild oat “seeds” we are referring to intact seeds, not to “caryopses.” **(B)** “Model system,” method of incubation of wild oat caryopses on *Fusarium avenaceum* strain *F.a.*1. (left) and untreated control (right). Wild oat M73 was chosen as the subject based on extreme dormancy level (Naylor and Fedec, 1978), allowing extended incubations.

In agro-ecosystems, weeds cause over $20 billion annually in crop damage and losses in the US (Pimentel et al., 2005). Seed dormancy and resistance to decay have a major economic impact due to the longevity of weed seeds in the “soil seed bank.” Weed seeds persist in the soil seed bank of agro-ecosystems in astonishing numbers often exceeding 10,000/m^2^ (Baskin and Baskin, 2006). Seeds commonly survive for years or decades, depending on the species and environment. Wild oat (*Avena fatua* L.), a model for our research, is shown in **Figure 1A** as the seed and dissected into caryopsis and hulls (lemma and palea). Wild oat has a longevity of 2–9 years *in situ*, depending upon environment and cropping system (Beckie et al., 2012); genetic diversity for dormancy in wild oat probably also contributes to variable longevity (Naylor and Fedec, 1978). There are few cases where targeting the dormant weed seed bank has been proven effective and economical (Davis, 2006). Therefore, ecological approaches to alter seed dormancy, viability, and/or longevity that promote weed seed decay could lead to novel biological control alternatives.

In proposing a “seed defense theory,” Dalling et al. (2011) state that seeds have four mechanisms of resistance to decay: “(i) physical barriers that render seeds impermeable to pathogens; (ii) endogenous chemical defenses of seeds; (iii) chemical defenses of beneficial seed–microbial associations; and (iv) rapid seed germination.” For seeds with physiological dormancy, Dalling et al. (2011) predicted that “microbial and chemical defenses, if present, will be arrayed on the exterior of the seed” and there are indeed reports of secondary chemical defenses (e.g., phenolics, tannins) associated with seed coats (Hendry et al., 1994; Gallagher et al., 2010). There are also reports of bacteria and fungi associated with seed surfaces that may contribute to seed defense (Chee-Sanford et al., 2006; Gallery et al., 2007, 2010; Chee-Sanford, 2008; Dalling et al., 2011; Long et al., 2014). Seeds with physical dormancy (impermeable seed coat or fruit wall) were predicted to rely on physical defenses against predators and pathogens (Dalling et al., 2011).

As a fifth defense mechanism, we hypothesize that seeds with physiological dormancy, such as wild oat, also possess enzyme-based biochemical defenses arrayed on the exterior of seeds. This proposed “biochemical” mechanism, based on defense enzymes, is distinct from the “chemical” mechanism which is attributed to lower molecular weight, non-protein secondary chemical defenses mentioned above. Consistent with this hypothesis, and as discussed in more detail below, we have shown that wild oat caryopses possess a defense enzyme, polyphenol oxidase (PPO), that is (1) induced by *Fusarium* seed-decay strains, (2) released from the caryopsis surface following challenge by *Fusarium avenaceum* strain *‘F.a.*1’ and (3) processed and activated, possibly by a protease (Anderson et al., 2010; Fuerst et al., 2011) following *F.a.*1 challenge. Furthermore, preliminary results suggest that three additional defense-response enzymes, peroxidase, oxalate oxidase, and chitinase, are induced by *F.a.*1 challenge. Our objectives here are to (1) review literature relevant to the seed defense enzyme hypothesis, especially information about PPO, but also some background on peroxidase, oxalate oxidase, and chitinase, (2) summarize our research on defense enzymes in cereals, specifically PPO induction in wild oat, (3) present an analysis of transit and signal sequences of seed PPOs of wheat (*Triticum aestivum* L.), and (4) present experimental questions implied by this new hypothesis. Seed longevity in the soil is not a major concern in wheat because wheat has relatively little dormancy and germinates rapidly, the result of domestication and plant breeding. However, our knowledge base for the biochemistry and genetics of defense enzymes in wheat, especially PPO and peroxidase, is far more extensive than in wild oat. Much of the information obtained in wheat may apply not only to wild oat seeds but also to seeds of many other species, and may therefore provide tools for understanding biochemical and molecular signaling in seed-pathogen interactions.

## CHARACTERISTICS OF PPO AND SEED DEFENSE ENZYMES

In order to be maximally effective in promoting seed longevity, PPO and other potential seed defense enzymes would need to be extrinsic (on or near the seed surface), bound or slowly released, durable, and capable of expressing activity after years of survival in the soil. PPO and peroxidase appear to possess many of these properties in wheat, in which they are primarily associated with the outer surface, as indicated by their high levels in the bran fraction when milling wheat (Fraignier et al., 2000; Rani et al., 2001). *In vitro* studies indicate that PPO and peroxidase are heat-tolerant (Vadlamani and Seib, 1996) and oxalate oxidase is tolerant of heat, protease, and detergents (Liang et al., 2001). Wheat PPO is predominantly present as an insoluble enzyme (Fuerst et al., 2006), perhaps available for activation and release by mechanisms discussed in this paper. The “whole kernel” PPO assay that we have utilized extensively (AACC International, 2000; Anderson and Morris, 2001; Fuerst et al., 2010) is indeed based on the extrinsic property of PPO on the wheat kernel. We have also observed that peroxidase and oxalate oxidase activities are readily measured by the same method in wheat (Fuerst, unpublished data), also implying their presence on the outer surface. Genetic studies are also consistent with PPO contributing to defense on the extrinsic surfaces of the seed: PPO activity and darkening of seed coat were strongly associated in recombinant inbred lines of pinto bean (*Phaseolis vulgaris* L.; Marles et al., 2008) and PPO was strongly associated with dark color of lemma and palea in *Setaria* spp. (Till-Bottraud and Brabant, 1990).

Polyphenol oxidases, peroxidases, and oxalate oxidases appear to have many properties in common that are relevant to seed defense. Isoforms of PPOs, peroxidases, and oxalate oxidases (1) are considered to be plant defense enzymes, (2) are involved in metabolism of oxygen and reactive oxygen species, (3) carry out the synthesis of low molecular weight defense compounds, (4) contribute to cross-linking of the extracellular matrix, (5) are considered heat-stable, and (6) are found on the exterior of seeds (Mayer, 2006; Dunwell et al., 2008; Almagro et al., 2009; Jerkovic et al., 2010). However, differences among these three enzymes include their metal co-factors: copper for PPO, manganese for oxalate oxidase, and iron for peroxidase, as well as their complementary roles in oxidative stress metabolism. For instance, oxalate oxidases generate hydrogen peroxide, and peroxidases use hydrogen peroxide as a substrate.

Perhaps the most compelling case for the extrinsic localization of defense enzymes was presented by Jerkovic et al. (2010). Proteomic studies demonstrated a great abundance and diversity of defense proteins in microdissected bran layers of wheat. Such proteins include the four enzyme types that we discuss here, PPO, peroxidase, oxalate oxidase, and chitinase; enzymatic activities of these enzymes were also demonstrated in water-soluble proteins from pericarp tissue and/or whole grains.

### HOST DEFENSE RESPONSES

Polyphenol oxidases are commonly associated with plant defense (Mayer, 2006; Constabel and Barbehenn, 2008). In many plant tissues, increased abundance of PPO transcripts in response to wounding and plant defense-related hormones such as systemin, salicylic acid, and jasmonates suggest that PPO genes are induced as part of a general defense response in plants (Constabel et al., 1998; Mayer, 2006; Flurkey and Inlow, 2008). PPOs are induced in incompatible (resistant) plant-pathogen interactions, including tomato *Fusarium wilt*, wheat head blight, potato late blight, and potato bacterial wilt (Mohammadi and Kazemi, 2002; Ramamoorthy et al., 2002; Thipyapong et al., 2007; Poiatti et al., 2009). PPOs are also induced in compatible (susceptible) interactions, such as potato soft rot and in non-host interactions, such as bacterial spot of citrus in potato. A direct link of PPO with disease severity and herbivore growth was demonstrated in PPO over-expressing and anti-sense genotypes of tomato, in which susceptibility to disease and herbivory were closely linked to the increased or decreased foliar PPO activities, respectively (Thipyapong et al., 2007). This induction of PPOs by a broad spectrum of pathogens and the relationship between PPO activities and susceptibility to disease and herbivory suggests that PPOs are part of plant innate immunity.

Peroxidase, oxalate oxidase, and chitinase, in addition to PPO, are among the defense enzymes induced in roots and foliage during challenge with fungal pathogens, and all are usually encoded by multi-gene families (reviewed in Lane, 2002; van Loon et al., 2006; Hücklehoven, 2007; Dunwell et al., 2008). All four enzymes participate in cell wall-associated host defense (Hücklehoven, 2007), the first line of defense against pathogen invasion, and all are expressed in seeds (Lane, 1994; Leah et al., 1994; Laugesen et al., 2007; Beecher et al., 2012).

Peroxidase is induced in tomato leaves infected with *F. oxysporum,* f. sp. *lycopersici*, *Pseudomonas syringae* and other pathogens, and by wounding (Ramamoorthy et al., 2002; Thipyapong et al., 2007). The class III peroxidases involved in host defense are wound- and pathogen-inducible, but in tobacco, peroxidase activity is not directly regulated by the defense phytohormones jasmonic acid and salicylic acid (Hiraga et al., 2001). In contrast, host oxalate oxidases are induced during interactions with biotrophic pathogens but not by wounding (Dumas et al., 1995; Zhang et al., 2013), suggesting different regulatory pathways among PPO, peroxidase, and oxalate oxidase, at least in leaves.

Class III peroxidases are secreted plant proteins with a remarkable number of functions including cross-linking cell wall polymers and lignification (Passardi et al., 2005; Almagro et al., 2009; Cosio and Dunand, 2009). Their role in defense is due to strengthening cell walls and massive production of reactive oxygen species. Oxalate oxidases (‘germins’) are a component of defense signaling in cereals (Lane et al., 1993; Hurkman and Tanaka, 1996; Dunwell et al., 2008) and have dual defense activities including the catabolism of fungal-derived oxalic acid, a metabolite toxic to plants, and production of fungicidal levels of hydrogen peroxide (Lane, 2002). Chitinases hydrolyze polymers containing *N*-acetylglucosamine such as chitin found in fungal cell walls, and are often associated with antifungal activity (Yan et al., 2008; Grover, 2012).

### BIOCHEMISTRY

Polyphenol oxidases utilize molecular oxygen to catalyze the hydroxylation and dehydrogenation of phenolic compounds to form reactive *o-*quinones, which alkylate nucleophilic groups and self-polymerize to form dark-colored melanin polymers. Most plant PPOs are capable of oxidizing a broad spectrum of *o-*phenolics. Haruta et al. (2001) reported that catechol derived from phenolic glycosides was likely a substrate involved in PPO-mediated herbivore defense and Marles et al. (2008) indicated that flavonols and condensed tannins, potential PPO substrates, are associated with seed coat darkening, but few other *in planta* substrates are known (Constabel and Barbehenn, 2008). Most plant PPOs are sequestered as latent enzymes in the chloroplast whereas most PPO substrates are located in other subcellular compartments. Cell disruption by herbivores and pathogens would allow PPO and its substrates to co-mingle; PPO-mediated reactions would then be maximized if PPO is activated. Likewise, disruption of this compartmentation is what leads to browning reactions in many fresh and processed food products, the result of PPO-mediated melanin polymer formation (Yoruk and Marshall, 2003; Mayer, 2006).

Nascent, unprocessed preproteins frequently contain *N*-terminal peptide sequences of ~20–60 amino acids that facilitate protein translocation from the site of synthesis in the cytoplasm to the subcellular target. Proteins targeted for chloroplast and mitochondria contain ‘transit peptides’ and proteins targeted for secretion via the endoplasmic reticulum contain ‘signal peptides’ (Gutensohn et al., 2006; Imai and Nakai, 2010; Yan and Wu, 2014). Although chloroplast transit peptides have been most commonly associated with unprocessed PPOs, PPO signal peptides for secretion have also been identified and vacuolar localization has been demonstrated in two species, *Antirrhinum majus* L. (snapdragon) and *Populus trichocarpa* Torr. and A. Gray (Western balsam poplar; Tran et al., 2012). The presence of signal peptides suggests that targeting other subcellular locations such as the extracellular space, might play a role in defense near the seed surface.

Four potential mechanisms by which PPO may inhibit pathogens and herbivores include (1) toxicity and antimicrobial activity of quinone products, (2) reduced bioavailability of proteins and nutrients, (3) creating lignin-like physical barriers, and (4) participating in the production of reactive oxygen species (Constabel and Barbehenn, 2008).

Most unprocessed plant PPOs range from ~68–73 kDa, which contain an *N*-terminal transit peptide that is cleaved to produce a ~55–68 kDa “mature” protein during transport into the chloroplast (van Gelder et al., 1997). These mature forms of plant PPOs are often latent or only partially active and proteolytic cleavage of a *C*-terminal peptide generally produces an active ~37–44 kDa PPO (van Gelder et al., 1997; Mayer, 2006; Flurkey and Inlow, 2008). However, harsh *in vitro* treatments including detergents, solvents, chaotropes, and proteolysis are also known to activate latent forms of PPO (Steffens et al., 1994; Flurkey and Inlow, 2008; Fuerst et al., 2010). Thus, mature plant PPOs are able to tolerate extreme changes in their environment, which likely explains the apparent stability of PPOs on the surface of seeds.

### GENETICS AND REGULATION OF PPOs AND DEFENSE ENZYMES

Most PPOs are encoded by multigene families whose members exhibit organelle-, tissue-, and development-specific expression (Steffens et al., 1994; Thygesen et al., 1995; Anderson et al., 2006; Beecher et al., 2012; Tran et al., 2012). Tomato (*Lycopersicum esculentum* L.) harbors seven genes encoded at a single locus on Chromosome 8; one, PPO F, is associated with defense in the leaves (Thipyapong et al., 1997; Newman et al., 2011). Developing tubers of potato (*Solanum tuberosum* L.) express five PPO genes, similarly clustered on Chromosome 8 (Thygesen et al., 1995; Thipyapong et al., 1997). The clustering of PPO genes in tomato and potato are considered to reflect their evolutionary origin via gene duplication. Diversity in gene number, sequence, tissue specificity, and substrate specificity across plant species suggest that the PPOs have long-term roles in fitness, niche adaptation and/or adaptation to environmental factors (Tran et al., 2012).

The transcriptional regulation of PPO genes is implied from the spatial and temporal expression patterns of gene family members in wheat, tomato, and potato (Thygesen et al., 1995; Thipyapong et al., 1997; Anderson et al., 2006; Beecher et al., 2012). Seed-expressed members of the PPO, peroxidase, oxalate oxidase, and chitinase enzyme families, particularly those expressed in the seed coat and aleurone, are especially relevant to our seed defense hypothesis. At least four to five distinct PPOs are expressed in seeds (caryopses) of hexaploid wheat (Massa et al., 2007; Beecher et al., 2012). These seed-expressed genes are clustered on Chromosome 2 of all three of the A, B, and D ancestral genomes (Beecher et al., 2012). In addition, the wheat seed-expressed PPO genes and other PPO genes usually contain introns (Massa et al., 2007; Beecher et al., 2012; Tran et al., 2012). The promoter of tomato *PPO B* regulates a family member expressed in the ovule seed coat and endosperm and harbors *cis*-acting elements responsive to phytohormones (ethylene, jasmonic acid, and gibberellic acid), associated with seed expression, and cAMP signaling (Newman et al., 2011). However, there is limited knowledge about transcriptional regulation of PPO in the seed coats of cereals. Analysis of promoters of seed-expressed wheat and wild oat orthologs would be informative when whole genome sequence data are available.

Examples in which other defense enzymes are expressed in seeds include four peroxidase proteins in barley aleurone (Laugesen et al., 2007), chitinase in barley aleurone (Leah et al., 1994), and oxalate oxidase associated with seed germination (Lane et al., 1991; Berna and Bernier, 1997). Isoforms of all four defense proteins have been localized to the seed coat or extracellular matrix of soybean (Gijzen et al., 1993; Lane, 1994; Gijzen et al., 2001), suggesting their potential defense roles in seeds. Likewise all four proteins and enzymatic activities were demonstrated to be associated with the extrinsic bran layers of wheat (Jerkovic et al., 2010), as previously discussed.

## DEFENSE ENZYMES IN WILD OAT AND WHEAT

We hypothesized, above, that seeds possess enzyme-based biochemical defenses arrayed on or near the exterior of seeds as a mechanism of resistance to seed decay, contributing to seed longevity in the soil. Consistent with this hypothesis, caryopsis PPO activity was induced by three *Fusarium* strains, although activity was also inhibited by a *Pythium* strain (Fuerst et al., 2011). *Fusarium avenaceum* strain *F.a.*1 caused the most rapid decay and the greatest induction of PPO. Wild oat seed and components are shown in **Figure 1A**. When whole wild oat seeds were incubated on *F.a.*1, PPO activity was induced in the whole seeds as well as in the dissected components: the hulls (lemma and palea) and caryopses. The induction of PPO activity in the hulls was surprising for a non-living tissue; however, latent PPO forms may be activated on the hulls in the same manner as in caryopses, as discussed below. *F.a.*1 induction of PPO in caryopses was greater than in intact seeds, and occurred more rapidly as well. Therefore our subsequent work focused on the *F.a.*1-wild oat caryopsis ‘model system’ (**Figure 1B**). PPO activity of *F.a.*1-treated caryopses was readily washed off, whereas very little PPO activity could be leached from untreated caryopses. This led to a series of studies on “caryopsis leachates,” focusing on that part of the PPO that was activated by *F.a.*1.

**FIGURE 2:**
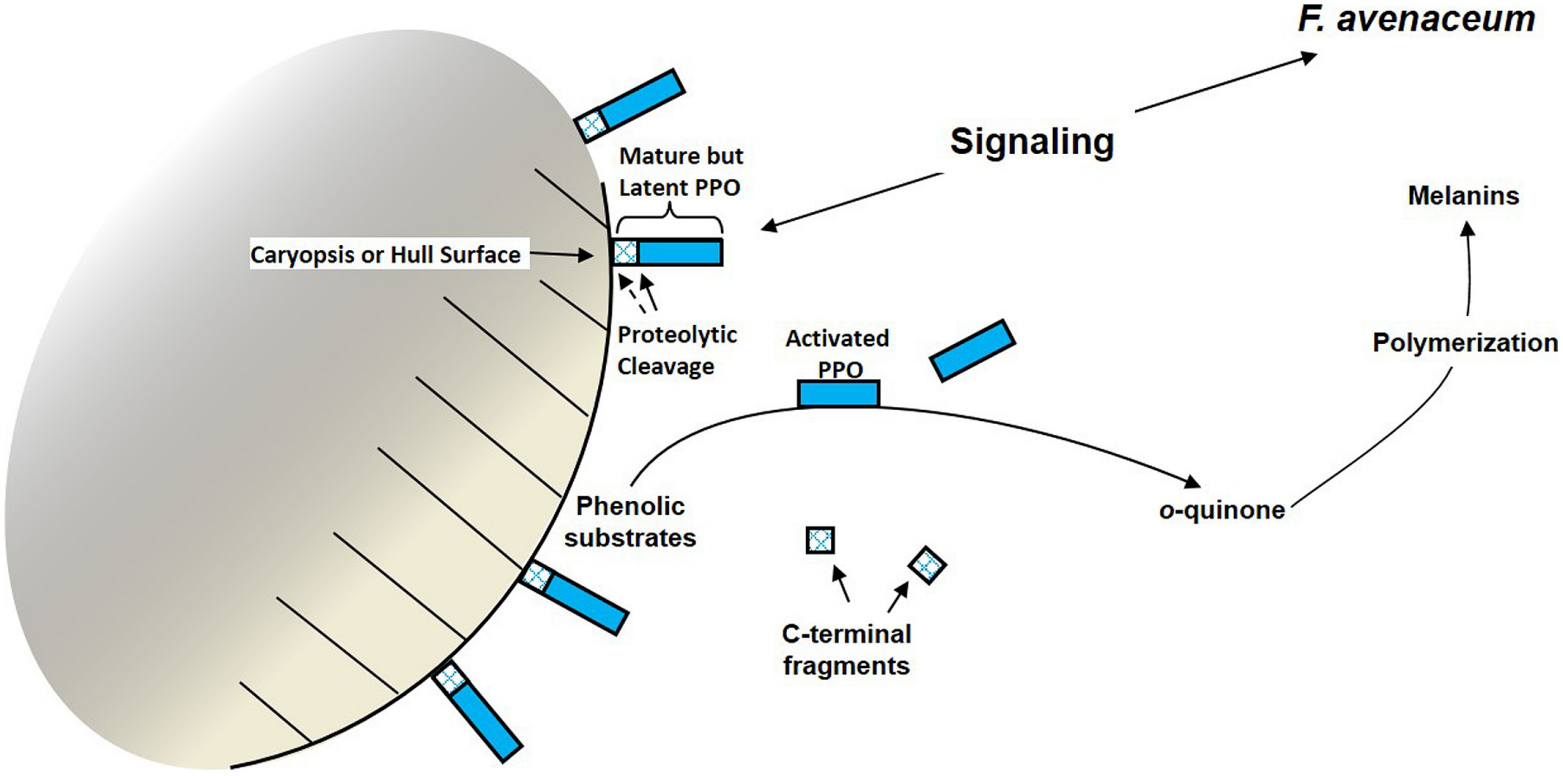
**Model for simultaneous activation and release of constitutive polyphenol oxidase (PPO) by proteolysis on the surface of a wild oat caryopsis following pathogen challenge.** Such a mechanism would not exclude PPO induction by other mechanisms, such as transcriptional induction. Both *o-*quinones and melanins are hypothesized to have anti-microbial properties (Constabel and Barbehenn, 2008).

We hypothesized that *F.a.*1-induced PPO activation in wild oat caryopses involved proteolytic cleavage that simultaneously activated and solubilized PPOs (**Figure 2**). This hypothesis was tested utilizing protein fractionation, immunoblots (westerns), and peptide sequencing (Anderson et al., 2010). The predominant form of PPO obtained from untreated wild oat caryopses and leachates was an inactive ~57 kD protein. Leachate from *F.a.*1-treated caryopses had a decreased abundance of the ~57 kDa PPO and increased abundance of PPOs ranging from ~52–14 kDa; these changes were associated with significant increases in both total activity and specific activity of PPO. The majority of PPO activity from untreated and *F.a.*1-treated caryopses was associated with a ~36 kDa protein. However, the *F.a.*1-treated caryopsis leachates also had PPO activity associated with ~25, and ~24 kDa proteins. Protein sequencing confirmed that the inactive ~57 kD and activated ~36 kD wild oat proteins were homologous to known PPO sequences. Results also suggested that activation of wild oat PPO involved the cleavage of a *C*-terminal peptide, consistent with proteolytic PPO activation in other plant systems (Flurkey and Inlow, 2008). These results support our hypothesis that mature, latent PPO is simultaneously activated and released into the environment, likely by proteolytic cleavage, as part of a defense mechanism during pathogen attack in wild oat caryopses.

Interestingly, peptide sequencing of the 25 and ~24 kDa proteins obtained from *F.a.*1-treated wild oat were most similar to a chitinase and oxalate oxidase, respectively (Anderson et al., 2010). Furthermore, separate preliminary studies demonstrated that *F.a.*1-treatment increased peroxidase activity in leachates approximately sixfold (Fuerst, unpublished data). Collectively, these observations suggested that multiple defense enzymes were induced by *F.a.*1 in wild oat caryopses.

## PPO TRANSIT AND SIGNAL PEPTIDES

The known and deduced amino acid sequences of PPO are informative for predicting plastidic or extracellular localization of mature proteins, hence distinguishing PPOs with potential for export and activation beyond the plasma membrane. Proteins harboring chloroplast transit peptides may not be mobilized without cell lysis, whereas proteins harboring extracellular signal peptides may be relevant to PPOs that reside on the surface of seeds or are transcriptionally induced as part of a defense system upon pathogen challenge. Whether constitutive and inducible defense activities can operate in parallel remains to be determined, but the possibility for this can be hypothesized if certain PPOs have chloroplast transit peptides and others have extracellular signal peptides. Several procedures for identifying different types of transit and signal peptides are available (Imai and Nakai, 2010). To test the hypothesis that wheat and other grasses encode PPO preproteins harboring signal peptides with potential for extracellular secretion, we conducted an *in silico* analysis of 12 wheat, one barley, and one *Brachypodium distachyon* protein sequences.

### MATERIALS AND METHODS

Analysis for the presence of extracellular signal peptides was conducted primarily using wheat proteins because genome sequence and transcriptome data were readily available for wheat but not for wild oat. Three web-based programs were used: SignalP 4.1 for prediction of signal peptides in eukaryotic proteins (Petersen et al., 2011), TargetP 1.1 for prediction of peptides for chloroplastic, mitochondrial or extracellular localization (Emanuelsson et al., 2000), and Signal-3L for prediction of signal peptides in plants (Shen and Chou, 2007). SignalP 4.1 predicted the secretory signal peptide cleavage site (*C*-score and *S*-score), and distinguished amino acids within a signal peptide from those in the processed protein (*Y*-score). The TargetP algorithm differentiated chloroplastic and mitochondrial transit peptides from signal peptides. TargetP reliability class (RC) values were assigned on a scale of 1–5, where 1 reflected the strongest prediction. The nominal RC values were based on differences between the most probable and next most probable output score. The Signal-3L output was qualitative (‘yes’ or ‘no’ for a signal peptide).

Amino acid sequences of ten seed-expressed PPOs from two wheat species (Beecher et al., 2012) were analyzed. A total of four additional monocot PPO sequences also were analyzed. One was a novel candidate PPO retrieved from the International Wheat Genome Sequencing Consortium [IWGSC] (2014) nucleotide sequence database. The PPO open reading frame was found at the 3′ end of IWGSC accession chr4DL_V3_ab_k71_14468617 using Blastn (Altschul et al., 1997) and the open reading frames of GenBank accessions JN632506 (PPO-A1h) and JN632507 (PPO-A2c) as query sequences. The deduced amino acid sequence of the IWGSC accession, designated IWGSC seg6A, was identified by alignments with two closely-related sequences from GenBank, a predicted protein from barley (AK358933; Matsumoto et al., 2011) and a PPO from *Brachypodium distachyon* (XM_003564319.1). Both the barley and *B. distachyon* sequences were included in the analyses. Finally, a second PPO genomic sequence from wheat cv. Chinese Spring was retrieved from GenBank (AB254806). The secreted protein cysteine proteinase RD21A (At1g47128) from *Arabidopsis thaliana* served as a control.

### RESULTS AND DISCUSSION

The SignalP 4.1 *C*-scores and *S*-scores for ten wheat seed-expressed PPO proteins ranged from 0.110-0.206 and 0.130-0.191, respectively. These were close to the ideal scores for a non-extracellular protein (**Table 1**). The SignalP *Y*-scores were also below the threshold for extracellular signal peptides. Absence of signal peptides was confirmed by Signal-3L (data not shown) and TargetP 1.1. A secretory signal peptide was strongly indicated as expected in the control cysteine proteinase RD21A, using the SignalP (**Table 1**) and Signal-3L analyses. Chloroplast transit peptides were predicted to occur in all ten wheat seed PPOs by TargetP 1.1 (**Table 1**). Chloroplast transit peptide scores were 0.60 or higher, whereas mitochondrial targeting peptide scores were below 0.52. The RC values were 2–4, reflecting intermediate prediction reliability values (1 = strongest prediction). The difference in RC values between the wheat PPO and the *Arabidopsis* control might reflect a bias in the TargetP algorithm for *Arabidopsis* proteins. As expected, cysteine proteinase RD21A was not predicted to have a chloroplast or mitochondrial targeting sequence.

**Table 1:** *In silico* analysis of potential chloroplast transit peptide sequences in ten wheat seed-expressed polyphenol oxidases.

		SignalP^1^				TargetP^2^			
Allele	Accession	*C*	S	Y	Prediction	cTP	mTP	SP	RC
PPO-A1h	JN632506	0.115	0.181	0.323	no SP	0.832	0.473	0.004	4
PPO-A1f	EU371654	0.115	0.181	0.323	no SP	0.833	0.472	0.004	4
PPO-A2c	JN632507	0.206	0.191	0.358	no SP	0.655	0.201	0.004	4
PPO-A2b	HQ228149	0.206	0.191	0.358	no SP	0.903	0.235	0.006	2
PPO-B2a	HQ228150	0.168	0.133	0.173	no SP	0.838	0.132	0.009	3
PPO-B2c	JN632508	0.167	0.135	0.202	no SP	0.764	0.326	0.005	3
PPO-D1a	EF070149	0.112	0.130	0.166	no SP	0.903	0.235	0.006	2
PPO-D1b	EF070150	0.136	0.183	0.339	no SP	0.848	0.524	0.004	4
PPO-D2a	HQ228152	0.110	0.191	0.357	no SP	0.598	0.265	0.004	4
PPO-D2b	HQ228153	0.131	0.121	0.204	no SP	0.781	0.132	0.00	4
At1g47128^3^	AY133781	0.625	0.763	0.972	has SP	0.010	0.013	0.993	1

*^1^The *C*-score and *S*-score predict signal peptide cleavage sites; the *Y*-score distinguishes amino acids within a signal peptide from those in the processed protein. Low values are associated with non-secreted proteins.*

*^2^High values predict chloroplastic transit peptide (cTP), mitochondrial transit peptide (mTP) or signal peptide (SP). The reliability class (RC) values were on a scale of 1–5, where 1 is the strongest prediction.*

*^3^The secreted cysteine proteinase At1g47128 from *Arabidopsis thaliana* served as a control.*

For the four additional monocot PPOs, SignalP, general for eucaryotes, predicted signal peptides for wheat AB254806 and *Brachypodium* XM_003564319.1 but not for the barley AK358933 or the wheat IWGSC seg6A proteins (**Table 2**). This contrasted with results from TargetP, specific for plant proteins, which predicted that the wheat IWGSC, barley, and *Brachypodium* PPO proteins carried extracellular signal peptides, but predicted that wheat AB254806 had a chloroplast transit peptide. Signal-3L (data not shown) predicted that all four proteins carried signal peptides. Our overall interpretation of these results favors the TargetP interpretation, which is both specific for plants (unlike SignalP) and clearly delineated chloroplast vs. mitochondrial transit peptides, whereas Signal-3L appeared less stringent in identifying signal peptides.

**Table 2:** *In silico* evaluation of potential signal peptides in four monocot polyphenol oxidases.

	SignalP^1^					TargetP^2^			
Accession	Plant	C	S	Y	Prediction	cTP	mTP	SP	RC
IWGSC seg6A	Wheat	0.232	0.769	0.336	no SP	0.006	0.079	0.794	2
AK358933	Barley	0.233	0.819	0.364	no SP	0.005	0.066	0.935	1
XM_003564319	*Brachypodium distachyon*	0.273	0.861	0.458	has SP	0.010	0.037	0.973	1
AB254806	Wheat	0.298	0.789	0.425	has SP	0.932	0.056	0.042	1
At1g47128^3^	*Arabidopsis*	0.625	0.763	0.972	has SP	0.010	0.013	0.993	1

*^1^The C-score and S-score predict signal peptide cleavage sites; the Y-score distinguishes amino acids within a signal peptide from those in the processed protein. Low values are associated with non-secreted proteins.*

*^2^High values predict chloroplastic transit peptide (cTP), mitochondrial transit peptide (mTP) or signal peptide (SP). The reliability class (RC) values were on a scale of 1–5, where 1 is the strongest prediction.*

*^3^The secreted cysteine proteinase At1g47128 from *Arabidopsis thaliana* served as a control.*

Although most plant PPO sequences contain chloroplast transit peptides, some PPO sequences contain *N*-terminal signal peptides for extracellular secretion, as previously discussed. Our results here are consistent with those generalizations, and indicate that signal peptides are probably present in at least one PPO preprotein sequence for each monocot species, wheat (IWGSC seg6A), barley (AK358933), and *Brachypodium* (XM_003564319.1). The non-chloroplast targeting may include extracellular secretion, a localization possibly related to the apparently extrinsic nature of PPO in wheat and wild oat caryopses (Fuerst et al., 2010, 2011).

## FUTURE RESEARCH

We hypothesize that seeds with physiological dormancy possess extrinsic enzyme-based biochemical defense mechanisms that contribute to seed longevity and survival in the soil. This hypothesis is based upon substantial but very specific evidence of PPO induction in dormant wild oat by the seed decay isolate, *F.a.*1, preliminary observations that peroxidase, oxalate oxidase, and chitinase may also be part of this defense response, and evidence for the extrinsic localization of defense enzymes especially as reported in wheat (Jerkovic et al., 2010). It is not known how broadly this mechanism applies in nature, and many questions remain, including: (1) In the specific case of PPO induction by *F.a.*1 in wild oat, is only constitutive (i.e., latent, mature) PPO activated by protease (**Figure 2**), or are active processes involved in this induction such as *de novo* PPO transcription and translation? (2) Does the protease come from the seed, or pathogen? (3) Are such defense enzymes widely present in seeds of other plant species including species with both physiological and physical dormancy? (4) Are seed defenses only induced by specific microorganisms, or more generally induced by a diversity of microorganisms including both seed decay pathogens and non-pathogenic microorganisms? (5) Do such enzymes actually contribute to seed defense and longevity in the soil and in the field? (6) Can knowledge of seed defenses and seed-microbe interactions be developed as a technology to enhance management of undesirable species, i.e., to promote the decay and decline of the weed seed bank? With so many unanswered questions, it is clear that this is a noteworthy opportunity for significant fundamental and applied research on enzyme-based biochemical and molecular seed defense mechanisms.

## Conflict of Interest Statement

The authors declare that the research was conducted in the absence of any commercial or financial relationships that could be construed as a potential conflict of interest.
